# A balance between meaningfulness and risk of harm – frail elderly patients’ perceptions of physical activity and exercise – an interview study

**DOI:** 10.1186/s12877-020-01868-2

**Published:** 2020-11-23

**Authors:** Kristina Åhlund, Birgitta Öberg, Niklas Ekerstad, Maria Bäck

**Affiliations:** 1 Department of Physiotherapy, NU Hospital Group, Trollhättan, Sweden; 2 Department of Health, Medicine and Caring Sciences, Division of Prevention, Rehabilitation and Community Medicine, Unit of Physiotherapy, Linköping University, Linköping, Sweden; 3 Department of Research and Development, NU Hospital Group, Trollhättan, Sweden; 4 Department of Health, Medicine and Caring Sciences, Division of Society and Health, Linköping University, Linköping, Sweden; 5 Department of Occupational Therapy and Physiotherapy, Sahlgrenska University Hospital, Gothenburg, Sweden

**Keywords:** Exercise, Multimorbidity, Physical activity, Qualitative

## Abstract

**Background:**

There is growing evidence of the benefits of physical activity and exercise for frail elderly patients with comorbidity. In order to improve participation in physical activity and exercise interventions, there is a need to increase our understanding of the patient’s perspective.

**Aim:**

The aim of this study is to explore the perceptions of physical activity and exercise among frail elderly patients with a severe comorbidity burden.

**Method:**

Face-to-face, in-depth interviews were conducted with eighteen frail elderly patients with a severe comorbidity burden, median age 85.5 years (min-max 75–94). The interviews were transcribed verbatim and analyzed according to content analysis inspired by Krippendorf.

**Results:**

An overall theme, defined as “Meaningfulness and risk of harm in an aging body” was identified, followed by three main categories, labeled *physical activity in daily life, goals of physical activity and exercise* and *prerequisites for physical activity and exercise,* and eight sub-categories.

**Conclusion:**

This study suggests that, in frail elderly patients with severe multimorbidity, physical activity and exercise is a balance between what is perceived as meaningful and the risk of harm. Patients perceived aging as an inevitable process that they needed to accept and gradually adapt their physical activities in daily life to match. As patients said they were unclear about the benefits and risks of exercise and referred to their previous life and experiences when describing physical activity and exercise, it is likely that the communication relating to this within the healthcare system needs to be further developed To promote physical activity and exercise to maintain or improve physical fitness in this frail population, healthcare providers need to use extended, personalized information to tailor the type of physical activities, goals and prerequisites for each patient.

**Supplementary Information:**

The online version contains supplementary material available at 10.1186/s12877-020-01868-2.

## Background

As the proportion and numbers of older people in society is increasing, more people are living with chronic diseases and frailty [1]. Frailty is regarded as one of the main risk factors for disability in aging. It is characterized by reduced physical reserves, which is reflected by vulnerability and it is strongly associated with institutionalization and mortality [2–5]. The physical phenotype of frailty is defined by Fried et al. [6] and it includes the following components: unintended weight loss, low muscle strength, poor endurance, slow walking speed and reduced physical activity. A person is defined as frail when three or more of these criteria are present. If one or two criteria are present, a pre-frail status is identified.

Physical fitness is defined as “*a set of attributes that is either health or skill related”,* e.g. cardiorespiratory fitness and muscle strength [7]. It is well known that poor cardiorespiratory fitness and muscle strength are associated with increased mortality in healthy individuals [8, 9] and it was recently found that these components of physical fitness also had an impact on mortality in frail elderly individuals with a severe comorbidity burden [10]. A reduction in physical fitness will increase the relative effort required to perform activities in daily life. In frail elderly individuals, where physical reserves are already reduced, a decrease in physical fitness may lead to difficulty maintaining independence in activities, such as climbing stairs, shopping and rising from a chair [11].

Physical activity and exercise are often used interchangeably, but it is important to note that they are not synonymous. Physical activity is defined as *“any bodily movement produced by skeletal muscles, which results in energy expenditure”* [7]. It is a complex behavior basically comprising most of the things we do in life and can be sub-divided into the domains of occupational, leisure, transportation and household activities [12]. Exercise is *“a subset of physical activity that is planned, structured, repetitive and purposeful in the sense that the improvement or maintenance of physical fitness is the objective”* [7]. Sedentary behavior is any waking behavior characterized by energy expenditure of ≤1.5 metabolic equivalents (METs), while in a sitting, reclining or lying position [13].

Older people are generally less physically active than younger adults [14, 15]. They have been shown to spend more time sitting and are mainly engaged in lower-intensity activities and rarely in activities of high intensity, compared with younger adults [16, 17].

Exercise is thought to be first-line therapy in frail elderly individuals when it comes to improving physical fitness, slowing deterioration and maintaining independence [18, 19]. Several systematic reviews conclude that structured, individualized prescribed exercise is beneficial in frail elderly individuals with regard to various outcome measurements, such as mobility, muscle strength, balance and rate of falls, cognitive function and quality of life [20–22]. There is no “golden standard” for exercise programs in this patient group, but multicomponent exercise interventions including resistance and aerobic exercises, as well as balance and flexibility exercises, have been recommended [23].

However, in frail elderly individuals with severe disability and comorbidity, studies including exercise interventions are rare. Difficulties due to recruitment, a high drop-out rate and problems related to transportation, among others, mean that these patients are often excluded from trials [24]. Excluding elderly individuals with substantial disability and comorbidity, who are seen every day in emergency medical hospitals, results in poor generalizability to a clinical population of frail elderly individuals [25].

Known barriers for exercise in frail elderly individuals are related to poor health and a perceived need for rest. Moreover, the patient’s attitudes and expectations of the exercise intervention and problems related to transportation to the exercise facility are important aspects related to participation and are described in the literature [26–28]. A systematic review [14] of both quantitative and qualitative research highlighted problems related to a lack of evidence relating to barriers and motives for exercise in the group of frail elderly individuals with severe comorbidity, as their perceptions may differ from those of less frail community-dwelling older people. Future studies of frail elderly patients with substantial comorbidity and disability are needed, as this group appears to be able to make obvious gains from increasing physical activity and participating in exercise programs [29]. To succeed, we first need to increase our understanding of the patients’ perceptions of physical activity and exercise, in order to tailor interventions according to their needs.

### Aim

The aim of this paper is to explore the perceptions of physical activity and exercise among frail elderly patients with a severe comorbidity burden.

## Method

The reporting of data in this study follows the consolidated criteria for reporting qualitative research (COREQ) [30].

### Design

This is a qualitative study with semi-structured interviews analyzed according to inductive content analysis inspired by Krippendorf [31] and refers to a descriptive and pragmatic way of interpreting text, without previous theory or excessive elements of abstraction.

### Setting

This study was derived from the research project “Is the treatment of frail elderly patients effective in an elderly care unit (TREEE)” which was conducted in the NU hospital group in western Sweden during the time period 2013–2015 and has previously been described [32].

### Patients

The inclusion criteria for the TREEE study were patients aged 75 years or older, assessed as frail according to the FRESH screening instrument [33] and in need of in-hospital medical care. FRESH is derived from the physical phenotype of frailty. Patients in acute need of organ- specific in-hospital care, i.e. related to acute myocardial infarction or stroke, were excluded. In the present study, patients with cognitive and communicative impairment were also excluded. To capture representative perceptions of physical activity and exercise, a strategic sampling procedure [31] was performed with the aim of including both men and women with frailty and varying functional status, living in both urban and rural settings. The intention was to include 15–20 participants, as this was considered appropriate in order to capture both common patterns and unique variations in relation to this context. The patients who fulfilled the criteria for participation and were considered to represent different views of the studied phenomena, were given informed consent consecutively out from the inclusion in the TREEE-study.

In this population of frail elderly patients with a severe comorbidity burden, it was reasonable to expect a certain drop-out rate. Twenty-three patients gave their informed consent to participate, of which 18 finally participated in an interview (9 women; median age 85.5 (min-max 75–94 years). The patients’ comorbidity burden was considered severe, with a median Charlson’s comorbidity index of 6 (5–11). The most common comorbidity diagnoses were hypertension (72%), cardiovascular disease (67%), renal failure (61%) and heart failure (56%). For the characteristics of the study population, see Table 1.

**Table 1:** Characteristics of the study population (*N* = 18)

Age, years, median (min-max)	85.5 (75–94)
Gender, female n (%)	9 (50%)
Frailty, FRESH score, median (min-max)	3 (2–5)
CCI, median (min-max)	6 (5–11)
Living alone, n (%)	10 (56)
Home service, n (%)	6 (33%)
Assistive device indoors (walker/crutches)	12 (67%) (9/3)

*CCI* Charlson’s comorbidity index, *FRESH* FRail Elderly Support researcH group

### Procedure

All the patients were enrolled and gave their informed consent in connection with an acute hospital care episode. Three months after discharge from hospital, each patient was called to schedule the time and place for an interview, by the author (KÅ). The interviews were performed in a location chosen by the participant (e.g. private room at the hospital or the patient’s home). If a patient’s relative was present during the interview, he or she was asked to remain silent and not to interact during the interview. A semi-structured interview guide was developed with the emphasis on the patients’ perceptions of physical activity and exercise, their goals and needs, perceptions of exertion, perceived barriers/facilitators and information from health care (additional file). Each interview began with the question *“How has your body functioned, since your discharge from hospital?”.* Follow-up questions were used with the aim of further deepening the dialogue. Four pilot interviews were performed to test the interview guide and interview technique. The interview technique was adjusted and improved by authors KÅ and MB to enable the patients to provide more detail in their narratives with follow-ups such as “Could you please tell me more” and “How did you experience it”. The pilot interviews were included in the analysis. The interviews lasted for a median of 28 min (range 20 to 43) and were tape recorded and transcribed verbatim.

### Analysis

The first step in the analysis included reading the interviews several times to obtain a sense of the whole. From the text, meaning units that captured the core intent were identified and labeled with a code. The codes briefly described the content of the sentence and were created with regard to the context and purpose. Codes with similar content were sorted into categories that reflected the patients’ perceptions of physical activity and exercise. According to Krippendorff [31], a category should be exhaustive and mutually exclusive. That meant that no data that corresponded to the purpose could be excluded for lack of an appropriate category, but there could be several sub-categories. The analysis procedure creating codes, sub-categories and categories was made close to the text in a manifest manner. In the last step of the analysis, the underlying meaning was abstracted and linked together to create a theme, representing the latent content.

The authors, KÅ and NE, enrolled patients in the study and author KÅ conducted the interviews. In the analysis process, KÅ and MB independently performed a preliminary analysis and the results were compared and, when necessary, discussed, until consensus was reached. After this, the results of the preliminary analysis were presented to and discussed in the same manner with BÖ and NE. None of the authors was involved with the patients’ care and rehabilitation. The first author (KÅ) works as a physical therapist and researcher. MB and BÖ are both experienced physical therapists and researchers used to qualitative methodology. NE is a physician and experienced researcher in the field of frail elderly individuals. KÅ and NE were both involved in the TREEE study, meaning that they met most patients included during the in-hospital episode to perform tests according to the study protocol [32, 34].

## Results

**Fig. 1:**
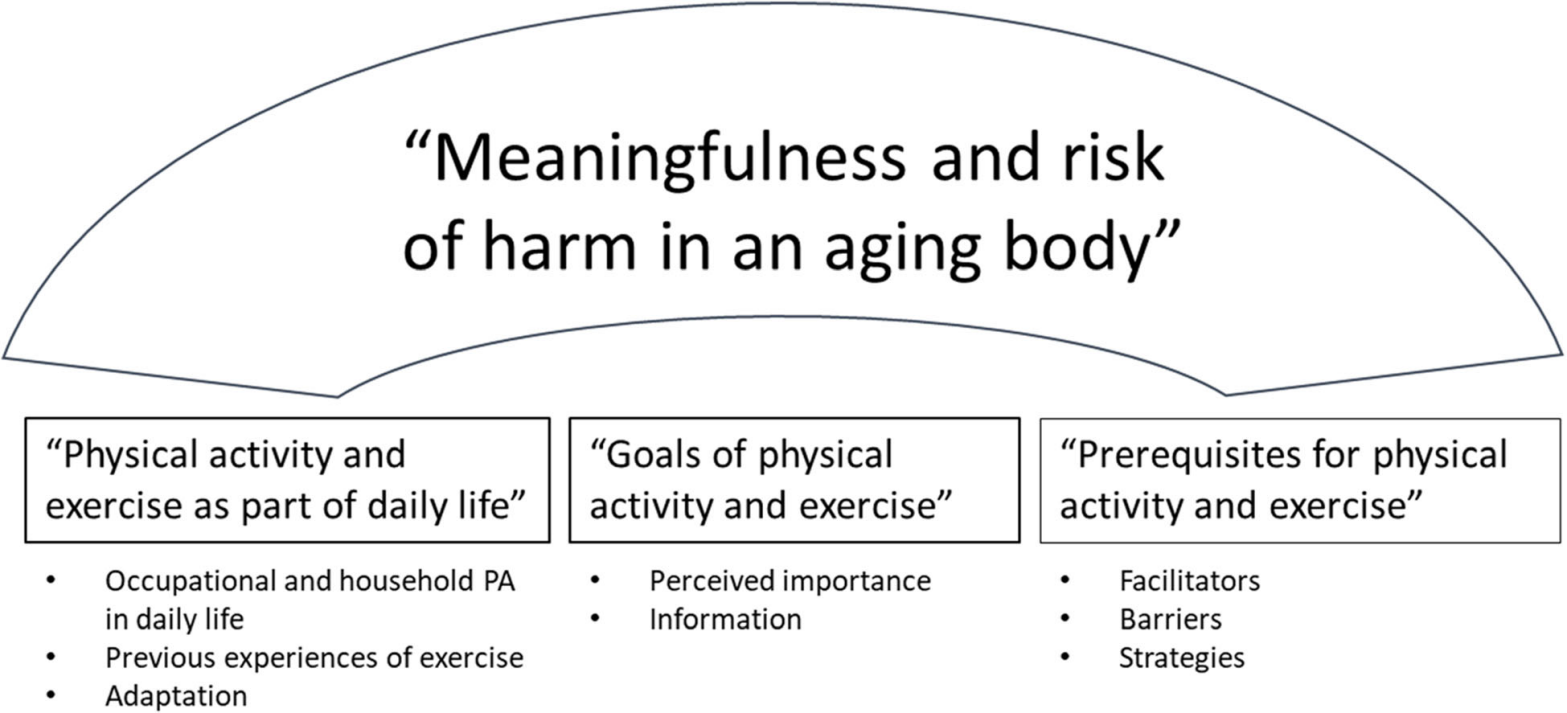
A summary of overall theme, categories and sub-categories. Abbreviations: PA Physical activity

The findings are presented as an overall theme, three main categories and eight sub-categories, see Fig. 1.

### Meaningfulness and risk of harm in an aging body (theme)

The overall theme “Meaningfulness and risk of harm, in an aging body” includes frail elderly individuals describing physical activity and exercise as a balance between what they perceive as meaningful to themselves and a risk of harm in relation to their aging body. Patients referred to physical activity by mentioning activities that they choose to perform in their daily lives, for example, cleaning, walking and gardening. They consider how to make it possible to perform physical activities in relation to their aging body and describe balancing benefits to maintain or improve capabilities and the risk of harm. Their choice of physical activities is based on reflections on personal goals, such as social interaction or maintaining independence. Strategies were described to make the activity feasible and to reduce the risk of falling – for example, the use of assistive devices.

### Physical activity and exercise as part of daily life (category)

Patients’ perceived physical activity and exercise as daily tasks often referred to as “work”. Their previous experiences during their whole life span colored their perceptions of physical activity and exercise. This includes childhood, occupational, leisure and household activities. In relation to their aging body, they gradually change their level and type of activity to adapt to their new situation.

#### Occupational and household physical activity in daily life (sub-category)

Patients described physical activity as part of their daily lives, for example, household activities and gardening. They could refer to their previous occupation and the work they used to do when they were employed.

*“It’s work (physical activity). We had a very large garden. We grew vegetables and everything so I had firewood for my spare time. I felled trees and lit the boiler. I was up and running all day.”*

*“No two days are the same. Sometimes I iron the laundry and sometimes I pick up and hang the washing.”*

*“I had the sort of job, you know, that meant I was always on the go. So, when I got home in the evening, I was tired… I was a school nurse. It was a great job. It was a wonderful job. With all the kids. I ran all the time, lots to do.”*

#### Previous experiences of exercise (sub-category)

When patients describe their thoughts, specifically about exercise, they relate to things they experienced when they were younger. A previous negative experience could have an impact on an individual’s present views of exercise.

*“I’ve actually been to a gym once ... but it’s many, many years ago. It wasn’t that easy, it really wasn’t. I would lie on one of those benches on my back and ... well, and do some exercises, whatever it was. I don’t remember why I decided to go there.”*

They also talked about positive experiences of having exercised, in the past.

*“We have four children and we previously did a lot of cross-country running, especially in the fall and springtime. Oh, it was a lot of fun... it’s 20 years, it must be 20 years ago.”*

*“I was very fit before. I worked very hard and I was strong… it was crazy. I worked hard and got a second breath and then there was nothing that could stop me ... and it didn’t hurt. I used to work in the forest.”*

#### Adaptation (sub-category)

The patients described the gradual adaptation of physical activity and exercise in daily life, due to changes in their physical function and their aging body over the years. The patients understood aging as an inevitable process they needed to accept and relate to.

*“I’ve been comfortable about being so old and I'm happy as long as I can do it… that I can move. Because that’s how I feel, just being and doing things myself…”*

*“Well, then I think about what I did before (exercise). When I played tennis, a lot ... Tennis isn’t possible anymore, because I have no balance in my body, in the same way. So, no, it’s not possible. In fact, I ended that 20 years ago.”*

### Goals of physical activity and exercise (category)

Patients described physical activity and exercise as ways of achieving goals in their daily lives, which they perceived as meaningful. Being physically active makes social interaction possible and enables the maintenance of independence, not to become a burden to their next of kin. They lacked information on physical activity and exercise from health care and expressed a desire for more information on how to perform physical activity and exercise. To promote increased knowledge through information, patients said that responsibility should be shared between healthcare and patients.

#### Perceived importance of PA and exercise (sub-category)

Patients described exercise as important for remaining independent in their daily lives, meaning being able to take care of themselves and not feeling that they are a burden on their family.

*“After I started doing these exercises in bed, I feel completely different. Not so helpless. Because I can do things myself. I get help with shopping, but I’m involved. For the last two weeks, I have been able to take part.”*

*“Obviously, that means a lot (physical activity). Otherwise, you become a burden, especially for your family. They would feel that mom has been stuck indoors. Yes, I think that’s the worst thing.”*

The patients said that performing exercise could be challenging and would not necessarily have any beneficial effects at their high age, in relation to the risks. They discussed the importance of performing activities properly and not exerting themselves.

*“Yes, I have many needs… but, since I have the attitude that I can’t do it now. I’m a 93-year-old man. After all, he can't exercise so much that he can get better.”*

*“… It wouldn’t be wrong (exercise), but it varies from individual to individual… Exercise naturally requires a lot more energy. And it may put pressure on your heart… I think you can gain a lot from exercising. But you have to do it properly.”*

Physical activity was described as social interaction. To walk to a meeting place or walk together with friends makes physical activity more meaningful.

*“I often go to the museum. It’s a sort of meeting place with exhibitions and concerts and so on. It quite simply offers you a place to meet. Many women are alone.”*

#### Information (sub-category)

The patients could not recall receiving information from healthcare professionals regarding physical activity and exercise, in relation to aging or disease. The patients said that they did not expect to receive information about physical activity or exercise, so they did not bring up the subject for discussion. After being given time to reflect on physical activity and exercise, the patients started to think about whether it could have been useful.

*“Yes, I wish they had talked about it to me. I may not do what I should. You don’t know if they don’t tell you. I would be grateful for this information. What to do and how to do it, but I haven’t heard it anywhere.”*

The patients also expressed a lack of knowledge about how to perform exercise. They said that they would like exercise programs based on personal needs and support. The also expressed a need for information to learn how to do it.

*“Yes, I can do it at home (exercise), but I have to get a program so I know what to do ... to give me a push, then I will do it ... If I get a program, I will have it next to me.”*

*“Well, it (exercise program) must be based on the patient’s personal needs …Yes, you have to start from the beginning. It is difficult to exercise, you know, without support.”*

### Prerequisites for physical activity and exercise (category)

The patients described different prerequisites for physical activity and exercise and mentioned facilitators that made performing physical activity and exercise easier, such as good assistive devices and easy transportation to the exercise facility. Barriers that prevented them from being physically active were also explained. Poor health was referred to as one important factor regarding the ability to be physically active. In relation to exercise, the patients expressed a need for an individualized setting. They also had different strategies so they could manage to do important activities.

#### Facilitators (sub-category)

Patients mentioned different external conditions that were important in order to facilitate their performance of physical activity and exercise. They described assistive devices as useful in activities of daily life and the distance and transportation to an exercise facility were factors, specifically important in order to facilitate participation in organized exercise.

*“I have been given some assistive devices. A chair so I can sit when cooking.*

*It is very good and makes things much easier.”*

*“Yes, I think that’s the way, where to go to exercise (facilitator)... Yes, how far it is to travel or walk. I think that's probably the main thing, if you have to get a taxi or if you have to walk, because now I am actually... going to stop driving.”*

#### Barriers (sub-category)

In relation to physical activity and exercise, patients perceived poor health, such as pain and perceived fatigue, as an important barrier. Other health-related problems were impaired balance and fear of falling, but fear of doing activities that could potentially harm them was also expressed as a barrier to physical activity and exercise.

*“You can’t manage. You go out and get pain in your legs and pain in your body. Many of my friends are in pain.”*

*“Of course, I walk, but I’m limited. My body is limited ... it’s hard to describe, you know. You simply cannot manage. You get tired and ... now I have to go and sit down, I say, and so that’s what I do.”*

*“I’ve fallen, you see. So, once you have fallen, it’s always there. It doesn’t go away. Fear, it stands in the way, I think…”*

#### Strategies (sub-category)

The patients said that they had to plan their activity carefully and they had their own strategies to minimize the risk of harm and to enable necessary activities.

*“You have to plan carefully, think and take many things at once (moving slowly). Like, when you have been in a room, maybe you have to take two or three things from there… you just have to remember it.”*

*“I think about the bathroom then (fall risk). Because that’s probably where people fall the most, I think ... we have a drying rack on the wall. So, I can grab it when I get out of the shower. I hold onto it and then I hold onto the washbasin. Because I have figured it out, that works well and so I can manage.”*

*“One day I increased the speed of the treadmill while walking (exercising). Then it went faster, and I got a little out of breath ... so, now I am on the low speed all the time and that works well ... well, at least I move my legs.”*

## Discussion

This study provides a deeper understanding of frail elderly patients’ perceptions of physical activity and exercise. Overall, the findings describe a balance between what the patients perceive as meaningful and the risk of harm, in an aging body. This includes aspects related to physical activity and exercise as part of daily life, goals for physical activity and exercise and the prerequisites needed to enable physical activity and exercise.

The results confirm previous knowledge that old patients may relate to physical activities in daily life, particularly household- and work-related activities, when describing their perceptions of physical activity and exercise [35] and when describing goals and barriers related to exercise [14, 28].

The novel aspect of this study is that it captures the patients’ perspective in a population of frail elderly patients with a severe comorbidity burden. There are indications that the oldest and most frail elderly patients experience different barriers and facilitators for physical activity and exercise than younger people and those with fewer disabilities [14]. In our study, patients described goals with physical activity and exercise in terms of being able to maintain independence. They also said that physical activity enables social interaction, both by providing opportunities to actually meet and by taking part in activities with other people, such as making walking possible. They regard physical activity as a tool to overcome loneliness. This can be contrasted to a study by Andreasen et al. [36], where experiences of daily life in frail elderly individuals, recently discharged from acute hospital care, were studied. They found that, the frailer the patients were, the more they experienced stressors and struggles in daily life, where disability, loneliness and inactivity became issues of special concern [36]. It is likely that, in old age, sufficient physical function is a cornerstone of independence in daily life and an important facilitator when it comes to maintaining autonomy related to social interaction.

It is, however, interesting that the patients in the present study described the gradual adaptation of physical activity in daily life, due to the natural process of aging. Physical deterioration has previously been regarded as a starting-point for feeling older [37]. Ekwall et al. [38] found that a sense of control and keeping everyday life as unchanged as possible was important for older adults, just after being discharged from hospital. Those patients said that physical ability related to their self-image and getting help to compensate for the deterioration, so that life could go on as usual, were important. As the patients in the present study were all frail with disabilities and a severe comorbidity burden, it could be hypothesized that their process of deteriorating had been ongoing for a longer period of time. They had gradually adapted to their new situation and accepted that their prerequisites for physical activity and exercise had changed due to their aging bodies.

Meaningfulness includes all the things that create or constitute meaning for a person [39] and the concept “Sence of Coherence” (SOC) [40] has been shown to be an important determinant of life-satisfaction. It highlights three components related to perceived SOC and meaning, which can be applied to the result of this study. According to SOC, *meaningfulness* is related to personal values and motivation. *Comprehensibility* is related to understanding the purpose of and knowing how to perform physical activity and exercise and is linked to what benefits and goals the patient sees. The patient must also see physical activity and exercise *manageable*, which means being able to handle the demands of the specific activity, e.g. pain, breathlessness or risk of falls related to illness and an aging body.

In the present study, perceptions that physical activity and exercise were deleterious and could increase the risk of harm were expressed during the interviews. It is likely that a poor knowledge of possible gains in relation to the risks influenced their thoughts. The patients mentioned that they did not expect any gains from exercising an old body which could outweigh the possible risks of over-exerting themselves or falling. These views can be compared with the results of previous research which, in a hospital setting, found that a multi-professional and holistic care approach including assessment and advice regarding physical function, was effective in preserving and improving physical fitness in frail elderly patients with a severe comorbidity burden [34].

Fear emanating from various origins, such as falls or deterioration, has previously been described as a barrier to exercise in the elderly [14]. In this study, comprising sometimes severely impaired patients, activities in daily life were perceived as exercise. Fear is part of their risk calculation and might reduce their willingness to perform daily activities.

Within the framework of health care, educational efforts designed to reduce fear and more clearly disseminate the positive effects of exercise are suggested [14, 28]. However, information has to be perceived as relevant to the older person’s needs and goals and tailored to their current situation. It is noteworthy that, in the present study, the patients were unable to recall having received information about physical activity and exercise from healthcare, despite regular contact with different healthcare providers. Good clinician-patient communication is associated with greater knowledge, increased adherence to treatment and self-care skills [41]. This indicates a need for improved routines when it comes to communicating aspects of physical function, physical activity and exercise to frail elderly individuals, in clinical health care.

Qualitative studies need to be carefully designed if they are to be trustworthy. One important aspect is the use of good sampling strategies. In this study, the population represents a rich variation of the studied phenomena by including frail elderly patients of various ages, genders and level of functioning.

To confirm authenticity, the patients were informed prior to the interviews that the interviewer was a physical therapist with no relationship to patient care. According to Krippendorff [31], the text obtains its meaning through the reader and it is a strength to have a knowledge of the subject. However, as a physical therapist in this particular context, it is important also to be aware of one’s preunderstanding related to physical activity and exercise. To avoid excessive interpretations in the analysis, the coding followed the text as closely as possible and only in the last step was interpretation allowed and a theme was created. To achieve good credibility, the analysis was performed in an iterative triangulation process, until consensus was reached between all the authors.

## Conclusions

This study suggests that, in frail elderly patients with a severe comorbidity burden, physical activity and exercise is a balance between what is perceived as meaningful and the risk of harm. The patients perceived aging as an inevitable process that they needed to accept and gradually adapt their physical activities in daily life to match. As the patients said that they were unclear about the benefits and risks of exercise and referred to their previous life and experiences when describing physical activity and exercise, the communication relating to this within the healthcare system likely needs to be further developed. To enable participation in physical activity and exercise in this group of patients, healthcare providers need to use extended, personalized information to tailor the type of physical activities, discuss strategies to make participation possible and assist in formulating valuable goals, which make physical activity and exercise meaningful for the individual.

## Supplementary Information

**Additional file 1.** These were the questions used to guide discussions during the interviews.

## Data Availability

The interviews analyzed in the current study are not publicly available due to identifying patient data should not be shared. Informed consent was not obtained for publication of patient data. Upon reasonable request, de-identified data may be available from the corresponding author.

## Abbreviations

CCI: Charlson’s Comorbidity Index
COREQ: Consolidated Criteria for Reporting Qualitative research
FRESH: FRail Elderly Support researcH group
MET: Metabolic Equivalent
PA: Physical Activity
TREEE: Is the treatment of frail elderly patients effective in an elderly care unit
