# Supplementary material for: A balance between meaningfulness and risk of harm – frail elderly patients’ perceptions of physical activity and exercise – an interview study
Source: BMC Geriatr. 2020 Nov 23;20:490. doi: 10.1186/s12877-020-01868-2 (PMC7685538; doi:10.1186/s12877-020-01868-2)
Supplement: Supplementary file 1 — Additional file 1. These were the questions used to guide discussions during the interviews. [file 12877_2020_1868_MOESM1_ESM.docx]

# Additional file: Interview guide

These were the questions used to guide discussions during the interviews (DOC 14 kb).

(The questions are translated from Swedish)

1. How has your body functioned, since your discharge from hospital?
2. How do you experience moving and being physically active?
3. What does it mean to you, to be physically active?
4. - What do you think of, when I say physical activity? (in general)
   - Does physical activity and exercise mean different things to you?
5. What does physical activity and exercise mean to you today?
   – Is it different to what it was before?
6. Describe a situation, when you are physically active?
7. Describe a situation where you feel that you are physically trying to make an effort?
   - Then, how do you act?
   - Then, what do you think about it?
8. What effects do you think regular physical activity and exercise have for a person?
   - Are there benefits?
   - Are there disadvantages?
   - Are there differences depending on age?
9. Can you describe your personal needs of exercise?
10. Can you describe what advice concerning physical activity and exercise you have received from health care personals?
    -What information (would you like to have) do you need?
11. What would make you participate in an exercise program?
    - Are there facilitators? ... barriers?
